# An Ex Vivo and In Silico Study Providing Insights into the Interplay of Circulating miRNAs Level, Platelet Reactivity and Thrombin Generation: Looking beyond Traditional Pharmacogenetics

**DOI:** 10.3390/jpm11050323

**Published:** 2021-04-21

**Authors:** Alix Garcia, Sylvie Dunoyer-Geindre, Séverine Nolli, Jean-Luc Reny, Pierre Fontana

**Affiliations:** 1 Geneva Platelet Group, Faculty of Medicine, University of Geneva, 1205 Geneva, Switzerland; alix.garcia@unige.ch (A.G.); sylvie.geindre@unige.ch (S.D.-G.); severine.nolli@unige.ch (S.N.); Jean-luc.reny@hcuge.ch (J.-L.R.); 2 Division of General Internal Medicine, Geneva University Hospitals, 1205 Geneva, Switzerland; 3 Division of Angiology and Haemostasis, Geneva University Hospitals, 1205 Geneva, Switzerland

**Keywords:** miRNA, biomarker, cardiovascular disorders, platelet aggregation, thrombin generation

## Abstract

Platelet reactivity (PR), a key pharmacodynamic (PD) component of the action of antiplatelet drugs in cardiovascular disease (CVD) patients, is highly variable. PR is associated with occurrence or recurrence of thrombotic and bleeding events, but this association is modulated by several factors. Conventional pharmacogenetics explains a minor part of this PR variability, and among determinants of PR, circulating microRNAs (miRNAs) have been the focus of attention during these last years as biomarkers to predict PR and clinical outcomes in CVD. This being said, the impact of miRNAs on platelet function and the mechanisms behind it are largely unknown. The level of a set of candidate miRNAs including miR-126-3p, miR-150-5p, miR-204-5p and miR-223-3p was quantified in plasma samples of stable CVD patients and correlated with PR as assessed by light-transmission aggregometry and in vivo thrombin generation markers. Finally, miRNA target networks were built based on genes involved in platelet function. We show that all candidate miRNAs were associated with platelet aggregation, while only miR-126-3p and miR-223-3p were positively correlated with in vivo thrombin generation markers. In silico analysis identified putative miRNA targets involved in platelet function regulation. Circulating miRNAs were associated with different aspects of platelet reactivity, including platelet aggregation and platelet-supported thrombin generation. This paves the way to a personalized antithrombotic treatment according to miRNA profile in CVD patients.

## 1. Introduction

Platelets are involved in several processes, such as hemostasis, inflammation, cell proliferation and immune system modulation [1]. The exposition of the sub-endothelium following vessel injury induces platelet activation and triggers thrombus formation. A similar process is observed at the site of atherosclerosis, where platelets play a pivotal role in triggering acute thrombotic events upon plaque disruptions or even endothelial erosions [2,3]. The role of platelets in thrombus formation is mediated via several processes, including aggregation and thrombin generation occurring at the surface of activated platelets following exposition of prothrombotic phospholipids [4,5]. Platelet reactivity (PR), a key pharmacodynamic (PD) component in patients with cardiovascular diseases (CVD) treated with antiplatelet drugs, is highly variable and has been associated with thrombotic or bleeding events. Interestingly, randomized controlled trials on PR-tailored antiplatelet regimens have provided contrasting results. In clinical studies, PR is usually quantified using aggregation-based assays, and the impact of PR on coagulation is not monitored. PR has heritable determinants in both untreated healthy volunteers and patients treated with antiplatelet drugs [6]. Traditional pharmacogenomics approaches have thus far provided limited data on the causes of this heritable PR variability. Indeed, a handful of genetic variants explain 5–12% of the on-clopidogrel PR variability [7], and data are even more limited for aspirin [8]. Similar to clinical outcome studies, pharmocogenetic studies focused on aggregation-based assays or receptor activity as surrogates of PR. Other approaches are needed to better explore this yet unexplained heritable PR variability, including genetic processes and a more global PR phenotyping.

miRNAs are small, noncoding RNAs that regulate gene expression at the post-transcriptional level and may modulate cell biogenesis and function. Platelets contain mature miRNAs and precursor miRNAs originated from megakaryocytes. The ability of human platelets to repress gene expression was confirmed by measuring the activity of the RISC complex [9,10]. The vast majority of circulating miRNAs originates from activated platelets [11]. Circulating miRNA are mainly transported by microvesicles, where they are stabilized by argonaute [12]. Therefore, the circulating miRNA profile mirrors platelet miRNA content, and selected circulating miRNAs were shown to be correlated with the recurrence of ischemic events in CVD patients [13,14], suggesting that platelet-derived miRNAs could be used as biomarkers to predict cardiovascular outcome [15] or to tailor antithrombotic therapy [16].

Only a few studies have investigated the association of circulating miRNA levels with different aspects of thrombus formation and have been mainly focused on platelet function assays [13,17,18]. We previously evidenced the role of miR-126-3p in platelet-supported thrombin generation [16], suggesting that miRNAs may not only regulate the platelet aggregation process but also their ability to trigger fibrin formation. In this work, our aim was to expand those latter results and to correlate a set of platelet derived miRNAs with platelet reactivity using light transmission aggregometry (LTA) as well as ex vivo thrombin generation markers. An in silico analysis of predicted gene targets was performed to propose a mechanistic hypothesis.

## 2. Materials and Methods

### 2.1. Study Population

Patients were selected out of the multicenter ADRIE (Antiplatelet Drug Resistance and Ischemic Events; clinicalTrials.gov identifier NCT00501423) population described in detail elsewhere [19,20]. This study focused on patients treated only with aspirin (*n* = 191) [16]. Briefly, outpatients enrolled in ADRIE between June 2006 and December 2008 and with previous symptomatic documented ischemic atherothrombotic disease were included. The ADRIE study was approved by the Central Ethics Committee of the University Hospitals of Geneva (Geneva center) and the Ethics Committee of Montpellier Saint-Eloi (Béziers and Montpellier centers). Written informed consent was obtained from all patients included in the ADRIE study.

### 2.2. Blood Collection

Blood was collected by venipuncture in EDTA and citrated tubes. Plasma was obtained by centrifugation of whole blood at 2300× *g* for 15min at room temperature and was stored unthawed at −80 °C until analysis.

### 2.3. Platelet Function Evaluation

Platelet function was assessed on platelet rich plasma (PRP) by LTA on an 8-channel aggregometer (TA-8V, SD Medical, Heillecourt, France) using adenosine diphosphate (ADP) 5 µmol/L and 20 µmol/L (Sigma, St Louis, MO, USA), Horm collagen 1 µg/mL (Nycomed, Linz, Austria) or arachidonic acid (AA) 1 mmol/L (BioData Corps, Horsham, PA) [19,20].

Thrombin-antithrombin complexes (TAT) and prothrombin fragments F1+2 (F1+2) were quantified in citrated plasma samples using Enzygnost TAT micro and Enzygnostat F1+2 kits (Siemens Healthcare Diagnostics Products, Marburg, Germany), respectively, according to the manufacturer instructions [16].

### 2.4. Selection of Candidate miRNAs

The selection criteria for the miRNAs quantified in this study was based on their expression profile in platelets; miR-126-3p, miR-150-5p and miR-223-3p were amongst the most expressed miRNAs [21] (http://www.plateletomics.com/, version 1.0, accessed 2 April 2021), and they were consistently associated with platelet reactivity in clinical studies [13,17]. We also investigated miR-204-5p, which was first described by our group as associated with platelet reactivity [22]; this association was confirmed subsequently in an independent cohort [23]. These four miRNAs are referred to below as candidate miRNAs.

### 2.5. Plasma miRNA Extraction and Analysis

EDTA-anticoagulated plasma samples were processed as previously described [16]. After thawing, samples were centrifuged at 1000× *g* for 5 min at 4 °C. One milliliter Qiazol and five femtomoles of Caenorhabditis elegans miR-39 were added to 2 × 100 μL of each sample. MiR-39 was used as a spike-in control in order to assess the efficiency of the purification and reverse transcription procedures. miRNA isolation was performed using the miRNeasy mini kit according to the manufacturer’s instructions (Qiagen, Hilden, Germany).

The reverse transcription procedure was carried out using a fixed volume of 2 μL of the elute RNA, and the reverse transcription products were preamplified with the TaqMan Advanced miRNA cDNA Synthesis Kit (Applied Biosystem, Foster City, CA, USA). Real-time qPCR analysis was performed using Taqman advanced miRNA assays and Taqman Fast advanced Master mix (Applied Biosystem). PCR reactions were run on 7900HT SDS system (Applied Biosystems). miRNA samples with qPCR cycle threshold (Ct) above 35 were excluded from the analysis.

A literature review pointed out miR-16, miR-93, miR-106-5p and miR-484 as putative normalizers in plasma samples [24,25,26,27]. These four normalizers were tested in all samples of the present study, and the best panel was determined using geNorm algorithm [28]. The relative level of each miRNA investigated was then calculated according to the technique described by Kok et al. [28].

### 2.6. Plasma Sample Quality Control

To identify a possible contamination of plasma samples with residual platelets, red blood cells and white blood cells, a quality control was performed in 20% of randomly selected plasma samples.

A Western blot analysis was performed using an anti-ITGA2b (CD41) and anti-PTPRC (CD45) antibody, a specific marker of platelets and white blood cells, respectively. Briefly, 2 μL of plasma was diluted in 18 µL of PBS and boiled 15 min at 95 °C in reducing buffer. Samples were loaded on NuPAGE 4–12% Bis-Tris Gel 10 wells (Invitrogen, Carlsbad, USA) then transferred to nitrocellulose membrane. The membrane was blocked with non-fat dry milk 5% and labelled with primary antibody anti-ITGA2b (Sigma) and anti-PTPRC (Abcam, Cambridge, UK). An IRDye 680RD secondary antibodies antibody (LI-COR Biosciences, Lincoln, RI, USA) was used for detection on Odyssey Imaging Systems (LI-COR Biosciences).

The ratio of miR-451 (a hemolysis-sensitive miRNA) to miR-23a-3p (a hemolysis-insensitive miRNA) evaluated by qPCR reflects hemolysis and is currently used as a quality control [12,29]. A ratio below 7 indicates the absence of significant hemolysis in the plasma sample [12].

miR-39 was measured by qPCR. The standard deviation of Ct values was determined in order to monitor the variability of extraction and RT procedures.

### 2.7. Gene Ontology and Pathway Analysis

Three databases (miRANDA, TargetScan and PicTar) were used to determine the putative gene targets of selected miRNAs. Genes included in at least two independent databases were included for further analysis.

Gene ontology (GO, https://geneontology.org, accessed on 7 April 2020) is a tool used to characterize the association between genes within biological pathways. In this work, we selected several GO-related pathways involved in platelet reactivity (platelet aggregation and platelet-supported thrombin generation). The selected pathways were “aggregation”, “P2Y12”, “actin cytoskeleton”, “thrombin” and “calcium homeostasis”. The GO terms associated with these selected pathways were defined using Amigo 2 (http://amigo.geneontology.org/amigo, accessed on 7 April 2020, Table 1). The genes belonging to the selected GO-terms that were also predicted to be targets of candidate miRNAs were selected for further analysis. These targets were then represented in a network built using Cytoscape (https://cytoscape.org/ version 3.8.2, accessed on 28 August 2020). Finally, a literature review allowed the linkage of candidate miRNA targets present in GO terms and platelet function.

### 2.8. Statistics

Correlation analyses were performed using a Spearman test. Data were analyzed using GraphPad Prism 7 software (GraphPad Software Inc., San Diego, USA). All *p*-values < 0.05 were considered statistically significant.

## 3. Results

### 3.1. Plasma Sample Quality Controls

**Figure 1:**
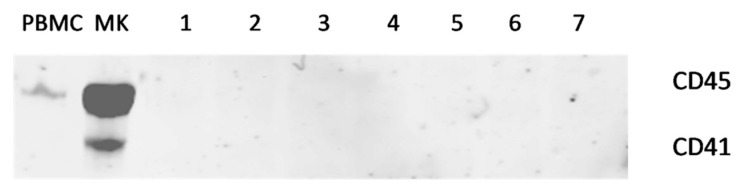
Representative Western blot of ITGA2b (CD41) and PTPRC (CD45) proteins observed in control samples (peripheral blood mononuclear cells (PBMC) and megakaryocytes (MK)) and in seven plasma samples randomly chosen to show the absence of residual cells in the plasma samples.

Western blot analysis in plasma samples revealed the absence of glycoprotein ITGA2b and PTPRC, indicating that no residual platelets and leukocytes were detectable in the samples. Representative examples are shown in Figure 1. The ratio of miR-451 to miR-23a-3p evaluated by qPCR was below 7 in all samples tested, confirming the absence of hemolysis.

In addition, qPCR analysis of cel-miR-39 added as a spike-in control before the extraction procedure showed a mean Ct value of 23.09, the coefficient of variation (CV) of the Ct values across the samples was 6.84%, indicating consistent extraction efficiency between the samples tested.

### 3.2. miRNA Reference Panel for the Normalization Procedure

We determined the most stable endogenous miRNAs that could be considered as reference for the normalization procedure in our study. The geNorm cut-off value of 1.5 [30] was applied, below which the endogenous miRNA is considered stable across samples and usable as a reference. miR-16, miR-93 and miR-484 were thus identified as the best combination of normalizers and were quantified in each sample (Table 2) [31]. The expression of the candidate miRNAs was then normalized against this set of stable miRNAs, as described elsewhere [28]. The relative expression of the miRNAs of interest is given in arbitrary units (AU).

### 3.3. Correlation between miRNA Level and Platelet Aggregation

Among the 191 plasma samples, miR-126-3p was successfully quantified in 183 samples, miR-150-5p, and miR-223-3p were quantified in 189 samples and miR-204-5p in 142 samples. Relative plasma miR-126-3p levels ranged from 0.5 to 9.2 arbitrary units (AU) [16], with a median value of 2. AU (IQR: 1.3–2.9). Plasma miR-150-5p levels ranged from 0.06 to 6.2 AU, with a median value of 0.7 AU (IQR: 0.4–1.2) [16]. Plasma miR-223-3p levels ranged from 0.04 to 3.04 AU, with a median value of 0.14 (IQR: 0.10–0.22), and plasma miR-204-5p levels ranged from 0.03 to 31.8 AU, with a median value of 0.65 (IQR: 0.25–1.36).

The circulating miRNA levels correlated with platelet aggregation results but with a different pattern for each miRNA. The magnitude of the correlation between platelet aggregation and miR-126-3p levels was the strongest using AA as agonist; the aggregation response to ADP correlated mostly with the other three miRNAs. Lastly, the aggregation response to collagen showed the highest correlation coefficient with miR-204-5p and miR-150-5p levels (Table 3).

### 3.4. Correlation between miRNA Level and In Vivo Thrombin-Generation Markers

There was a positive correlation between miR-126-3p or miR-223-3p levels and TAT or F1+2, while no correlation was observed between miR-204-5p or miR-150-5p levels and these latter thrombin generation markers (Table 3).

### 3.5. Gene Ontology Analysis

The miRNAs prediction databases respectively identified 146, 4156, 5820 and 3584 mRNA targets for miR-126-3p, miR-150-5p, miR-204-5p and miR-223-3p in at least one of the databases interrogated. When considering the predicted targets present in at least two databases, the numbers were narrowed down to 16, 232, 600 and 100 targets identified for miR-126-3p, miR-150-5p, miR-204-5p and miR-223-3p, respectively. The mRNA targets for miR-150-5p, miR-204-5p and miR-223-3p present in at least two databases were used for further analysis. Given the small number of common targets identified for miR-126-3p in 2 distinct databases, all the 146 putative targets predicted by at least one database were used for further analysis.

**Figure 2:**
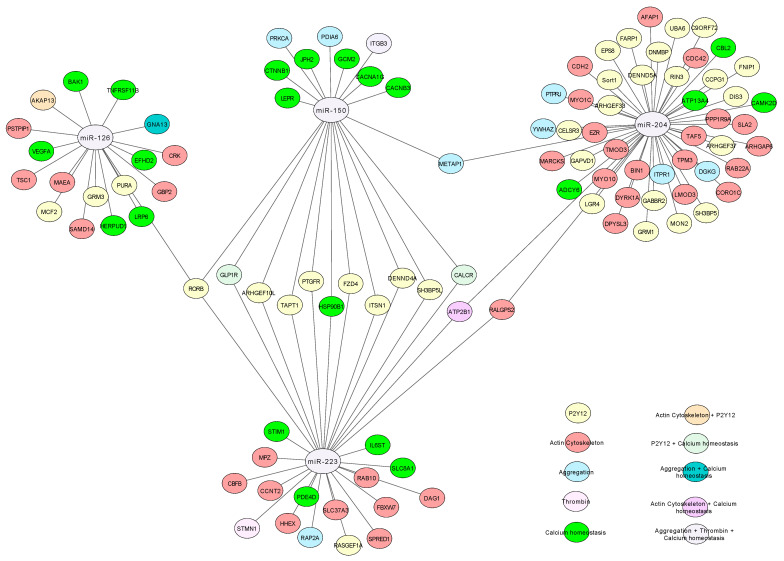
Network of miRNAs predicted targets and their involvement in platelet function-related pathways including aggregation (blue), P2Y12 (yellow), actin cytoskeleton (red), thrombin (pink) and calcium homeostasis (green).

The predicted targets of each miRNA were matched against selected gene ontology pathways including aggregation, P2Y12, thrombin generation, actin cytoskeleton and calcium homeostasis. Figure 2 depicts a network based on gene-gene interactions of the predicted genes of each miRNA. miR-150-5p and miR-223-3p were predicted to target 21 and 29 genes, respectively, including 10 common targets belonging to the P2Y12 pathway. The other genes potentially regulated by miR-150-5p and miR-223-3p were mainly a part of calcium homeostasis and actin cytoskeleton pathways, respectively. miR-126-3p was predicted to target the expression of 18 genes, including RORB, a nuclear hormone receptor that is also a putative target for both miR-150-5p and miR-223-3p. Finally, miR-204-5p was predicted to modulate the expression of 49 genes, including a majority of genes involved in actin cytoskeleton and P2Y12 pathway modulation. Like miR-150-5p, miR-204-5p was predicted to target methionine aminopeptidase 1 (METAP1) involved in G protein-coupled receptors (GPCR) signaling. Ral GEF with PH Domain and SH3 Binding Motif 2 (RALGPS2) known to modulate cytoskeletal organization and ATPase plasma membrane Ca2+ transporting 1 (ATP2B1) were putatively targeted by both miR-204-5p and miR-223-3p.

## 4. Discussion

Circulating miRNAs are predominantly derived from platelets and are often associated with platelet reactivity in a wide variety of clinical studies. However, the vast majority of these studies investigated healthy subjects or CVD patients on dual antiplatelet therapy (aspirin and an anti-P2Y12—mostly clopidogrel). Instead, we used an original approach in investigating patients treated with aspirin only, thus overcoming the heterogeneity of the response to clopidogrel mainly due to a variability of liver-dependent activation of the pro-drug, rather than to the variability of platelet physiology [32]. Up to now, studies on correlations between circulating miRNA levels and platelet reactivity were usually restricted to platelet aggregation assays. We previously demonstrated the role of miR-126-3p in platelet-supported thrombin generation [16], which is a crucial step in thrombus formation, and we now expand these results by evaluating the correlation of other candidate miRNAs with both thrombin generation markers and platelet aggregation in the ADRIE study.

Pre-analytical steps are of utmost importance in order to avoid contamination with other sources of miRNAs, such as residual platelets, which may be a major confounding factor in such association studies [17]. Both citrate and EDTA plasma samples may be used to evaluate circulating miRNA [33]. We rather used EDTA samples since its use is associated with a lower final calcium content and inhibits more profoundly the platelet activation that may occur during the collection process [17]. We paid particular attention to ruling out significant contamination of the plasma samples with platelets, red and white blood cells. Nevertheless, a contamination below the detection methods used in this study cannot be ruled out.

### 4.1. Association of Candidate miRNA with Platelet Aggregation

We measured the correlation between candidate miRNA levels and maximal platelet aggregation in response to the three agonists used in the ADRIE study, including collagen, AA and ADP. Collagen is considered a strong agonist that binds to platelet GPIaIIa and GPVI receptors and is dependent on amplification pathways tested with AA and ADP. AA induces the formation of TxA2 under the successive action of enzymes, including COX-1, the target of aspirin. TxA2 binds to its specific G protein-coupled receptor (TP) and triggers platelet activation. Although AA-induced platelet activation is usually drastically inhibited by aspirin, the relevance of this agonist for identifying COX-independent pathways was evidenced in a 700-patient study [34] and its importance was illustrated in another study of our group showing that AA-aggregation in aspirin-treated patients contributed equally to other agonists and to the construction of a platelet reactivity index [35]. ADP is a soluble agonist released by platelet dense granules and mediates its effect via two GPCR, P2Y1 and P2Y12. Our results show that the four candidate miRNAs correlated with platelet aggregation response to at least two different agonists.

miR-223-3p level was positively, although weakly, correlated with platelet aggregation induced by collagen and ADP. This latter correlation is surprising since miR-223-3p targets P2Y12, and therefore a negative correlation would appear more logical. However, miR-223-3p impacts the expression of several other genes, including glucagon-like peptide 1 receptor (GLP1R), an in silico-predicted target of this miRNA. Of note, the presence of GLP1R in platelets is controversial and a matter of debate since several groups [36,37,38] have demonstrated the presence of GLP1R in platelets by FACS, Western blot and qPCR, while another group [39] failed to detect GLP1R expression using a ddPCR method. GLP1R belongs to the P2Y12 pathway (Figure 2), and activation of this receptor inhibits platelet aggregation induced by ADP, collagen or thrombin [40]. We would therefore speculate that downregulation of GLP1R expression by miR-223-3p could increase platelet aggregation induced by ADP and collagen, despite a possible decrease of P2Y12 receptors. Our results are in line with those of Kaudewitz showing a positive correlation between miR-223-3p and plasma levels of platelet activation markers such as P-selectin and PF4 in the general population (Bruneck cohort) [41].

miR-126-3p levels correlated with AA- and ADP-induced platelet aggregation. These results are in line with the results of Kaudewitz and co-workers [41] who showed that the inhibition of miR-126-3p in mice decreases AA-induced platelet aggregation. They also showed a positive correlation between miR-126-3p levels and P-selectin secretion [41], which was subsequently confirmed in a model using human hematopoietic stem cells [42]. Our in silico investigations showed that A-kinase anchoring protein (AKAP13), a member of actin cytoskeleton and P2Y12 pathways, may be downregulated by miR-126-3p. AKAP13 is known to be a regulator of cAMP, leading to the inhibition of calcium release, G protein activation, adhesion, granule release as well as aggregation [43,44,45].

We also evidenced a positive association between miR-204-5p levels and platelet aggregation induced by AA, ADP and collagen, supporting the known association between this miRNA and platelet reactivity in patients with acute coronary syndrome [23]. Our in silico strategy identified several gene targets related to P2Y12 and actin cytoskeleton pathways. Among those, CDC42, a validated target of miR-204-5p [46], is of major interest. Indeed, we demonstrated that miR-204-5p regulates the expression of CDC42 that in turn induces an increase of platelet reactivity in a model using human-derived platelet-like structures [47].

### 4.2. Association of Candidate miRNAs and In Vivo Thrombin Generation Markers

Platelets promote thrombus formation via an aggregation process and thrombin generation triggered by the exposition of procoagulant phospholipids on their surface. We acknowledge that the in vivo thrombin generation markers used in the present work are not specific of platelet-supported thrombin generation. However, whereas in current models the initiation phase of thrombin generation takes place on tissue factor-expressing surfaces, the propagation phase occurs on surfaces containing procoagulant phospholipids, mostly represented by activated platelets and platelet-derived microvesicles [48]. Moreover, using human-derived cells, we previously showed that overexpression of miR-126-3p in megakaryocytes was associated with an increased generation of procoagulant platelet-like structures and that miR-126-3p was associated with in vivo thrombin generation markers [16]. Continuing from our previous work, this study identified miR-223-3p as an additional platelet-derived miRNA associated with these markers in CVD patients. This suggests that both miRNAs could have a synergetic effect and support a miRNA profile as a predictor of the ability of platelets to generate thrombin at their surface.

Our in silico strategy did not allow us to identify any direct target of miR-126-3p belonging to the thrombin generation pathway as defined in GO. However, AKAP13, a predicted target of miR-126-3p, regulates calcium homeostasis, a cornerstone in the generation of procoagulant platelets [5]. We also identified Stathmin 1 (STMN1), a validated target of miR-223-3p [49] that could be involved in the regulation of platelet-supported thrombin generation by regulation of annexin V exposure [50]. These findings suggest that miR-223-3p could increase the platelet-supported thrombin generation via down regulation of STMN1.

**Figure 3:**
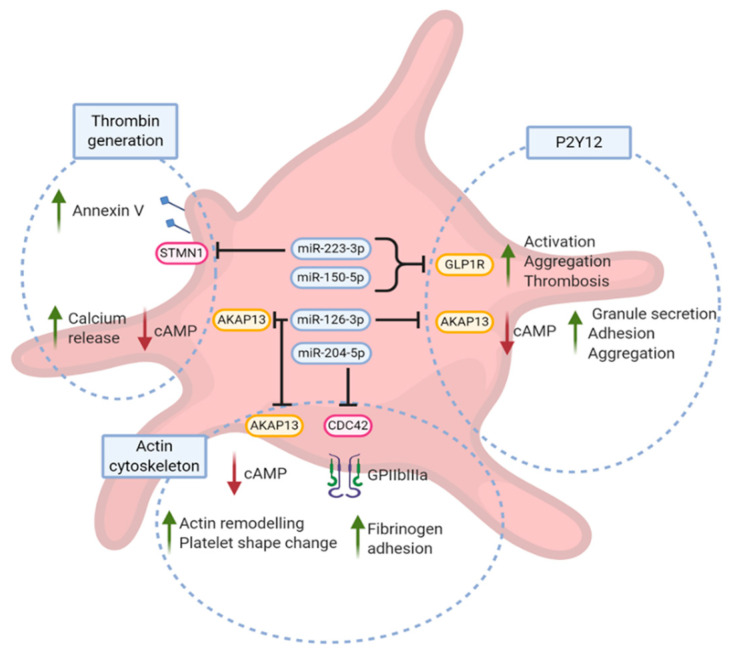
Putative associations between miRNAs and platelet function. The predicted target genes of candidate miRNAs were matched against GO-pathway genes (open circles) involved in platelet function (P2Y12, thrombin generation and actin cytoskeleton). Red boxes: experimentally validated gene targets; yellow boxes: in silico-predicted gene targets.

The main functional effect of miRNAs on platelet physiology is likely to occur in megakaryocytes, where the majority of proteins are produced. However, the impact of miRNAs within platelets should not be neglected since they contain the necessary machinery to process miRNA precursor [9] and that platelet miRNAs regulate important functions such as platelet shape change, granules secretion, and platelet activation [10]. The impact of miRNA in platelet function is also supported by a recent study directly modulating miRNA content within human platelets [51]. The present work suggests that platelet-derived miRNAs may differ in their ability to regulate platelet reactivity, including the aggregation process and the platelet-supported thrombin generation capacity. However, the magnitude of the correlations between miRNA levels and platelet function assays were relatively weak, although significant, but are in line with the magnitude of the correlation observed by other groups [41,52]. This is expected since miRNAs have a role of fine-tuning with a limited regulation of gene expression and suggests that a single miRNA could not be an accurate biomarker of platelet reactivity. Therefore, we speculate that a panel of several miRNAs would allow better prediction of platelet function. Of note, antiplatelet drugs have an impact on circulating miRNAs [53], and the correlation found in cardiovascular patients may not be true in healthy subjects. Figure 3 provides a model of the putative association between the selected studied miRNAs and platelet reactivity. miR-150-5p and miR-223-3p are both predicted to target GLP1R mRNA that mediates ADP-induced aggregation through a P2Y12-dependent mechanism. miR-204-5p regulates platelet function, at least in part, via CDC42 regulation. Finally, miR-126-3p may regulate platelet aggregation through actin cytoskeleton and P2Y12-related pathways mediated by AKAP13. The causative link between miR-126-3p and thrombin generation has not yet been established but may occur via modulation of calcium release by AKAP13. In addition, miR-223-3p could promote thrombin generation via STMN1 mRNA downregulation. Finally, only a few of the in silico predicted miRNA targets described in this work are validated using luciferase reporter gene assays, and most genes in Figure 3 are therefore speculative. Therefore, further studies should be carried out to validate the remaining targets as well as to study the underlying mechanisms.

Altogether, we identified the relationship between four miRNAs and platelet reactivity, including platelet aggregation and thrombin generation in stable CVD patients taking aspirin. Our original approach and data add to the current knowledge from traditional pharmacogenetics studies that were up to now very limited in aspirin treated patients and were restricted to a few CYP450 variants in clopidogrel treated patients. Since circulating miRNA levels reflect platelet activation status in real time, miRNA profiles could be used as biomarkers to predict distinct facets of platelet function. It could also prove useful in tailoring antithrombotic therapy, favoring low dose anticoagulant in patients with a miRNA profile associated with an increased platelet-mediated thrombin generation.

## Figures and Tables

**Table 1:** Gene ontology (GO) terms related to platelet aggregation and thrombin generation pathways.

Pathways	GO Terms
Aggregation	Platelet aggregation
	Platelet activation
	Regulation of platelet aggregation
P2Y12	G protein-coupled adenosine receptor activity
	ADP receptor activity
	G protein-coupled receptor activity
	Guanyl-nucleotide exchange factor activity
	G protein-coupled purinergic nucleotide receptor activity)
Thrombin generation	Thrombin-activated receptor activity
	Thrombin-activated receptor signalling pathway
	Fibrinogen binding
	Blood coagulation common pathway
Actin cytoskeleton	Actin cytoskeleton
Calcium homeostasis	Vacuolar calcium ion homeostasis Golgi calcium ion homeostasis
	Cellular calcium ion homeostasis
	Mitochondrial calcium ion homeostasis
	Mitochondrial, calcium ion homeostasis
	Endoplasmic reticulum calcium ion homeostasis
	Smooth endoplasmic reticulum calcium ion homeostasis
	Regulation of cytosolic calcium ion concentration
	Circadian regulation of calcium ion oscillation
	Bone remodeling

**Table 2:** Plasma miRNA reference panel determination using geNorm algorithm. The geNorm M value was used to measure expression stability. An M value below 1.5 indicates that miRNA is stable across samples and usable as a reference.

miRNA	geNorm M Value
miR-106-5p	1.018
miR-93	0.926
miR-484	0.874
miR-16	0.760

**Table 3:** Correlations between circulating miRNA levels and platelet aggregation and thrombin generation markers in stable cardiovascular patients. * The association between miR-126-3p and miR-150-5p with thrombin generation markers are derived from Zapilko et al. [17]. AA: arachidonic acid; ADP 5: adenosine diphosphate 5 μM; ADP 20: adenosine diphosphate 20 μM; F1+2: prothrombin fragment 1 + 2; NS: not significant; TAT: thrombin–antithrombin complex (Spearman correlation test).

Agonists	miR-126-3p		miR-204-5p		miR-150-5p		miR-223-3p	
	Rho	*p*-Value	Rho	*p*-Value	Rho	*p*-Value	Rho	*p*-Value
AA	0.210	0.004	0.183	0.029	0.079	0.282	0.058	0.431
ADP 5	0.074	0.321	0.184	0.028	0.132	0.071	0.015	0.837
ADP 20	0.148	0.046	0.158	0.06	0.257	<0.001	0.166	0.023
Collagen	0.144	0.053	0.279	0.001	0.260	<0.001	0.178	0.014
F1+2	0.329 *	<0.001 *	0.173	0.040	0.124 *	0.092 *	0.224	0.002
TAT	0.182 *	0.014 *	0.115	0.174	0.046 *	0.533 *	0.281	<0.001

Spearman correlation color code:
NS<0.20.2–0.3>0.3

## Data Availability

The data are available on reasonable request to the corresponding author.
